# Parasite infestation influences life history but not boldness behavior in placental live-bearing fish

**DOI:** 10.1007/s00442-020-04795-6

**Published:** 2020-11-03

**Authors:** Andres Hagmayer, Andrew I. Furness, Bart J. A. Pollux

**Affiliations:** 1 Experimental Zoology Group, Department of Animal Sciences, Wageningen University, 6708 WD Wageningen, The Netherlands; 2 Department of Ecology and Evolutionary Biology, University of California, Irvine, CA 92697 USA; 3 Department of Biological and Marine Sciences, University of Hull, Hull, HU6 7RX UK

**Keywords:** Matrotrophy, Parasites, Placenta, Poeciliidae, *Poeciliopsis retropinna*

## Abstract

Parasites can negatively affect the reproductive success of hosts. Placental species may be particularly susceptible, because parasite-induced stress during pregnancy could potentially influence embryo development. Here, we examine the consequences of a trematode infestation (black spot disease, BSD) for fetal development and adult behavior in 19 natural populations of the placental live-bearing fish species *Poeciliopsis retropinna* (Poeciliidae) in Costa Rica. First, we observed substantial variation in parasite infestation among populations which correlated with a number of local environmental conditions (elevation, river width, depth, and flow velocity). Furthermore, we observed substantial variation in parasite infestation among females within populations associated with maternal age and size. We found that the infestation rate significantly influenced embryonic development, with more heavily parasitized females producing smaller and worse-conditioned offspring at birth, possibly, because a costly immune response during pregnancy limits, either directly or indirectly, nourishment to developing embryos. Finally, a behavioral experiment in the field showed that the infestation rate did not affect an individual’s boldness. Our study indicates that in placental live-bearing fish parasite infestation leads to reduced embryo provisioning during pregnancy, resulting in a smaller offspring size and quality at birth potentially with negative implications for offspring fitness.

**Electronic supplementary material:**

The online version of this article (10.1007/s00442-020-04795-6) contains supplementary material, which is available to authorized users.

## Introduction

The life history of individuals describes how resources are allocated to different functions such as maintenance, somatic growth and reproduction (Roff 1992; Stearns 1992). Individuals have limited resources that must be competitively allocated to these different functions. This leads to trade-offs and a limiting set of possible life-history strategies (Braendle et al. 2011). Parasites, which are ubiquitous in natural populations (Bush et al. 2001), can act on these trade-offs and induce shifts in the optimum of life-history traits (Michalakis and Hochberg 1994; Sheldon and Verhulst 1996; Richner 1998). Therefore, studying the causes and consequences of parasite infestation is crucial for understanding the evolution of life histories.

Parasites can negatively affect the fitness of their host by directly or indirectly influencing their survival and/or reproductive success (Bush et al. 2001). Effects on survival can be the direct result of parasite-induced mortality. Soay sheep (*Ovis aries*) that are highly parasitized by gastrointestinal nematodes are less likely to survive the winter during periods of high overwinter mortality (Coltman et al. 1999). Moreover, lactating bighorn ewes (*Ovis canadensis*) were shown to be more heavily infested by lungworms compared to non-lactating ewes, and ewes that started to reproduce early in life suffered greater mortality from disease (Festa-Bianchet 1989). Lactation (Prentice and Prentice 1988) and parasite defense both impose energetic costs on the host (Sheldon and Verhulst 1996), suggesting that lactating bighorn ewes are more susceptible to parasites because of the conflicting energetic demands of milk production and parasite resistance (Festa-Bianchet 1989). Survival can also be indirectly impacted, for example by increasing the vulnerability to predators. Guppies (*Poecilia reticulata*) experimentally infected with a small number of cercariae of the digenean trematode *Diplostomum spathaceum* have been shown to be more susceptible to predation by brook trout (*Salvelinus fontinalis*) (Brassard et al. 1982). The increased susceptibility to predation was correlated with decreased swimming activity (measured as distance travelled per unit of time) of infected fish. Likewise, parasites can reduce the overall fecundity of hosts, either directly by affecting the nutritional status of hosts (e.g. Hurd 2001; Decaestecker et al. 2005; Tobler et al. 2005) or indirectly (e.g. by increasing offspring mortality; Brown and Bomberger Brown 1986; Møller 1990). If parasites reduce the host’s fitness, this can have profound implications for population dynamics of both hosts and parasites (Gulland 1995).

Hosts may modify their behavior in response to a parasite infestation (Barber et al. 2000). Boldness is one of the major personality axes in animals that may be affected by parasite infestation (Barber and Dingemanse 2010). Boldness, defined as the propensity of an animal to engage in risky behavior, has direct implications for fecundity and survival (Sih et al. 2004). For instance, increased boldness allows individuals to forage at higher rates, leading to increased growth and/or fecundity (Sih et al. 2004). However, increased boldness also increases the mortality risk from predators, and thus, individuals must balance the conflicting demands of feeding and predator avoidance (Sih 1980). Given that parasites can negatively affect an individual’s fecundity and survival, high levels of boldness might be favored, if the compensation of the fitness costs due to predation and parasitism is efficient (Kortet et al. 2010). For instance, in sticklebacks (*Gasterosteus aculeatus*), individuals parasitized by cestode larvae showed increased foraging activity and recovered more quickly following an attack with a heron model (Giles 1983). This increased activity and boldness was argued to compensate for the extra nutritional requirements caused by the parasite (Giles 1983). Thus, parasitism may play an important role in shaping aspects of animal personalities, such as exploration, activity, or boldness (Barber and Dingemanse 2010).

Black spot disease (BSD) is caused by a trematode parasite (*Uvulifer* sp.) that is commonly found in freshwater fish (Lane and Morris 2010). This trematode uses aquatic snails and fish as intermediate hosts, and piscivorous birds as the final host (Hoffman and Putz 1965; Lane and Morris 2010). The sexually mature trematode produces eggs in the intestine of the bird. The eggs develop into miracidia that are released by the bird through its feces. The miracidia invade snails where they reproduce asexually and develop into free-swimming cercariae. The cercariae penetrate the skin of fish and become encapsulated by the host’s tissue where they remain dormant until the fish is consumed by a piscivorous bird (Hoffman and Putz 1965; Tobler et al. 2007; Lane and Morris 2010). The penetration of the fish skin induces a melanic secretion by the host around the parasite, which forms externally visible black spots that are easily countable (Lively et al. 1990). Moreover, the penetration of the fish skin causes mechanical damage and hemorrhage, and the host’s induction of melanocysts that enclose the parasite is expected to be energetically expensive (Lane and Morris 2010; Cureton et al. 2011). In line with this, BSD was shown to reduce the body condition of smallmouth bass (*Micropterus dolomieui*) (Hunter and Hunter 1938) and juvenile bluegill sunfish (*Lepomis macrochiris*) (Lemly and Esch 1984). Likewise, infected females of the live-bearing mosquitofish (*Gambusia affinis*) avoided shoaling with infected individuals (Tobler and Schlupp 2008), and females of the amazon molly (*Poecilia formosa*) avoided infected males (Tobler et al. 2006), which suggests some costs of associating with infected conspecifics (but see *Poecilia latipinna* and *Poecilia mexicana* in Tobler et al. 2006). Notably, however, these studies were done on egg-laying (oviparous) or egg-carrying live-bearing (lecithotrophic viviparous) fish species. The potential implications of BSD for behavior, embryo development, and quality of offspring in placental live-bearing fish remain poorly understood.

Here, we study the consequences of BSD for life-history variation and boldness in a live-bearing fish species, *Poeciliopsis retropinna* (family Poeciliidae, Regan 1908), from Costa Rica. In this species, females transfer nutrients to their developing embryos via a ‘follicular placenta’, a structure that is analogous to the mammalian placenta (Pollux et al. 2009). Placentas form a physical interface between mother and fetus, allowing for intimate maternal–fetal interactions (e.g. respiration, nutrition, removal of waste products) that are crucial for normal (healthy) embryonic development. However, this intimate link also poses a risk, because maternal exposure to adverse environmental conditions (e.g. malnutrition, parasite infestation) may have unfavorable consequences for fetal development. For example, maternal parasite infestation can affect fetal growth in two non-mutually exclusive ways: (1) directly, through infestation of the developing fetus by parasites that can cross the placental barrier; and (2) indirectly, through the modification of maternal physiology or metabolism to such an extent that it interferes with fetal development. The influence of parasite infestation on embryo development has primarily been studied in mammals (e.g. Andrews and Lanzer 2002; Torrico et al. 2004; Gibney et al. 2008). However, placentas have evolved many times throughout the animal kingdom (e.g. Wourms 1981; Blackburn 2015; Wake 2015), including in the family Poeciliidae (Reznick et al. 2002; Pollux et al. 2014; Furness et al. 2019), yet the consequences of parasite infestation for offspring development, life-history traits, and behavior in non-mammalian placental lineages are currently insufficiently understood. *P. retropinna* has a particularly well-developed placenta (i.e. embryos can undergo an over 100-fold weight gain during gestation; Reznick et al. 2002) and is often found in well-defined populations (Hagmayer et al. 2020) making it an ideal system to study the consequences of parasitism in natural populations.

To study the causes and consequences of BSD in *P. retropinna*, we (1) quantify the intensity of parasite infestation by scoring the number of black spots on preserved adult female *P. retropinna*, (2) examine potential environmental predictors of parasite infestation among populations, (3) identify potential maternal predictors of parasite load within populations, and (4) relate parasite load within populations to maternal life-history traits (egg mass at fertilization, offspring mass at birth, proportion of egg and offspring fat, reproductive allotment, average brood size, fecundity, superfetation, and abortion incidence) to evaluate potential costs of parasitism. Finally, we (6) explore whether these costs influence an individual’s behavior by assessing its boldness (boldness score and hesitancy) in a field experiment. In doing so, our study sheds light on the importance of host-parasite interactions in shaping life histories and behavior in placental live-bearing fish.

## Materials and methods

### Study species and collection sites

*Poeciliopsis retropinna*, a live-bearing fish species in the family Poeciliidae, reaches a maximum standard length of approximately 80 mm. This species is found in freshwater streams of varying water velocity in Costa Rica and Panama (Bussing 2002). During gestation, *P. retropinna* females transfer nutrients to developing embryos via a placenta (Pollux et al. 2009). The degree of post-fertilization maternal provisioning in this species is extensive, with offspring increasing in dry mass more than 100-fold during gestation (MI = 117) (Reznick et al. 2002). Moreover, *P. retropinna* has superfetation, the ability to carry several broods at different developmental stages (Hagmayer et al. 2020).

During February and March 2017 and 2018, *P. retropinna* were collected at 19 different locations in the Rio Terraba and Rio Coto drainages in the province of Puntarenas, Costa Rica (Online Resource Table S1). Each location was characterized by measuring (1) elevation above sea level, (2) mean river width, (3) mean river depth, and (4) mean water velocity (Supplementary Methods 1.1). At each location, 5–37 adult females were collected using seine and cast nets, euthanized with an overdose of MS-222, and preserved in 5% formaldehyde.

### Laboratory measurements

Maternal standard length and the proportion of body fat were measured using established protocols (Supplementary Methods 1.2). The intensity of parasite infestation was quantified by counting the number of black spots on each preserved female. The ovaries were subsequently dissected to count the total number of embryos (i.e. fecundity), regressors (i.e. aborted embryos), broods at different developmental stages (i.e. superfetation), embryos in a given brood (i.e. brood size), and to determine the developmental stage and average dry mass of the embryos in a brood (Table 1). The developmental stages are based on morphological criteria described in Haynes (1995) and range from 0 (eggs at fertilization, no development) to 45 (fully developed embryos). Fecundity was calculated by excluding stage 0 eggs, since it was difficult to assess if they were fertilized. Instead, to ensure that all eggs in our study were fertilized, embryos at developmental stage 2, rather than 0, were defined as ‘eggs at fertilization’.

**Table 1:** Summary of maternal life-history traits

Maternal life-history traits	
Egg mass at fertilization	Dry mass of eggs at fertilization (i.e. developmental stage 2)
Offspring mass at birth	Dry mass of fully-developed embryos (i.e. developmental stage 45)
Proportion egg fat	Egg fat at fertilization divided by dry mass of eggs at fertilization
Proportion offspring fat	Offspring fat at birth divided by offspring dry mass at birth
Absolute reproductive allotment	Total dry mass allocated to reproduction (i.e. embryo dry mass, regressor dry mass, and placental dry mass)
Average brood size	Average number of embryos in a brood for a given mother
Fecundity	Number of embryos carried by a female counted across all broods excluding stage 0 embryos
Superfetation	Number of broods at different developmental stages
Abortion incidence	Number of regressors (i.e. aborted embryos) divided by the sum of the number of regressors and embryos

### Behavioral trials

Boldness was assessed on the 27th and 28th of February 2020 between 11:00 and 16:00 in a single population of *Poeciliopsis retropinna* from Rio Tinoco, Costa Rica. In the morning of each day, 32–35 *P. retropinna* were collected from closely located pools using a seine net and stored in a 120-L plastic bucket covered with a lid. The sample consisted of adults, defined as large (potentially pregnant) females and mature males (fully developed gonopodium present); immatures, defined as small (non-pregnant) females and males that did not have fully developed gonopodia; and juveniles, defined as fish < 2 cm. Pregnancy in this species is indicated by a dark gravid spot in the belly area. For the statistical analysis, however, we only used individuals that could be sexed (i.e. 40 adults and 21 immatures). To prevent the same individuals from being caught on different days, the same pools were not sampled twice. Prior to the behavioral trial, an individual was randomly selected from the bucket and placed into a glass container (30 × 20 × 2 cm) that minimizes bending movement of the fish. The fish was photographed from both sides and the top to (1) determine sex, (2) quantify parasite load, and (3) measure standard length using the image analysis software ImageJ (Abràmoff et al. 2004).

The experimental set-up to quantify boldness consisted of a dark plastic box (17 × 12 × 11.5 cm) with a non-transparent lid placed on the top and a trapdoor (7.5 cm wide and 9.5 cm high) in the front (Brown et al. 2005). A metal ring was placed underneath the box to form a D in a radius of 8 cm in front of the box (Online Resource Fig. S1). The box was positioned in approximately 15 cm of water at the edge of the pool from which the fish were collected on day 1. As a result, all fish were provided with the same experimental conditions, except that the fish collected at day 1 were released into an environment with which they were more familiar than the fish collected on day 2. We statistically accounted for variation in behavior between different days. Each fish was gently poured into the dark plastic box and allowed to acclimate for 2 min. A single fish was measured at a time. After the acclimation period, the trapdoor in the front of the box was opened and the fish was free to emerge and to swim into the pool. For each fish, both the time taken to emerge from the box after opening the trapdoor and the time to cross the metal ring was recorded. If the fish had not emerged from the box and crossed the metal ring after 10 min, we terminated the trial. Boldness was then defined as (1) the time taken for the fish’s snout to emerge from the box (boldness score), and (2) the time the fish took to cross the metal ring minus the time it took to emerge from the box (hesitancy) (Brown et al. 2005).

### Statistical analysis

All analyses were carried out in R v 3.6.3 (R Core Team 2020): mixed models were fitted in a Bayesian framework using the MCMCglmm package (Hadfield 2010). Convergence was assessed by visual examination of the traces and the autocorrelations of the parameter chain was checked to be less than 0.1. The priors, number of MCMC chains, iterations, burnin, and thinning are given in the Electronic Supplementary Materials. Negative binomial models were fitted using the glmmTMB package (Brooks et al. 2017).

To identify site-specific environmental sources of variation in black spot infestation, we modeled the proportion of parasitized individuals per sampling location in a series of generalized linear models using maximum likelihood and a logit link for the binomial-distributed response (Online Resource Table S2). Fixed effects in the full model included the elevation of the sampling location, mean river width and depth, as well as mean water velocity (all *z*-standardized). The models were ranked on the basis of Akaike’s information criterion adjusted for small sample sizes (AICc) (Burnham and Anderson 2002). However, collinearity among the environmental variables (Online Resource Table S3) causes difficulties in choosing the ‘best’ model, as several models each containing different (but correlated) predictors may provide similar fits (Freckleton 2011). Rather than selecting the ‘best’ model according to the AICc, we computed model-averaged parameters based on the models with ΔAICc < 2 (Grueber et al. 2011). Specifically, each parameter estimate was averaged over the models in which that predictor appears and was weighted by the summed weights of these models (Burnham and Anderson 2002). For the subsequent statistical analyses, we only used the data from locations where black spot disease (BSD) was found (at 16 out of 19 sampling locations; Online Resource Table S1).

To quantify individual sources of variation in black spot infestation, we fitted parasite load (number of black spots per female) as a function of the proportion of maternal body fat and standard length in a generalized linear mixed model using maximum likelihood and a log link for the negative binomial-distributed response. The negative binomial model accounts for over-dispersion arising from individual heterogeneity in parasite load. Sampling location (i.e. population) was fitted as random intercept accounting for spatial non-independence of observations.

The potential life-history consequences of black spot infestation were evaluated by fitting the maternal life-history traits (egg mass at fertilization, offspring mass at birth, proportion of egg and offspring fat, reproductive allotment, average brood size, fecundity, superfetation, and abortion incidence) as a function of parasite load (number of black spots per female) in a multivariate (generalized) linear mixed effects model allowing for the covariance between the residuals of all responses. Additional fixed effects included the proportion of maternal body fat, which is believed to be a good indicator of fish condition (Leips et al. 2013), and maternal standard length (Hagmayer et al. 2018). In the case of reproductive allotment, fecundity and superfetation, the developmental stage of the most-developed brood was fitted as an additional fixed effect to account for females early in the reproductive cycle. Sampling location (i.e. population) was fitted as random intercept accounting for spatial non-independence of observations.

To optimize normality and homoscedasticity of model residuals, reproductive allotment was ln-transformed, and abortion incidence, proportion of egg and offspring fat, and maternal body fat were arcsin square-root transformed. Fecundity and superfetation were fitted in generalized linear mixed effects models using a log link for the Poisson-distributed responses.

Behavioral responses to black spot infestation were evaluated by first modelling the probability of a fish emerging from the box and crossing the metal ring. For this, a binary variable indicting whether an individual emerged from the box and crossed the metal ring was fitted as a function of parasite load (number of black spots), sex, day, and standard length in a generalized linear model using maximum likelihood and a logit link for the binomial-distributed response. An additional predictor was the time difference (s) between the behavioural trial and capture (*z*-standardized). However, this effect was not significant (*χ*_1_^2^ = 0.560, *P* = 0.454) and thus excluded from any further analysis. Second, both the boldness score (the time taken to emerge from the box) and hesitancy (the time the fish took to cross the metal ring minus the time it took to emerge from the box) were each fitted as a function of parasite load (number of black spots), sex, day, and standard length in generalized linear models using maximum likelihood and a log link for the quasipoisson-distributed responses. In both cases, the time difference (s) between the behavioural trial and capture (*z*-standardized) was not significant (boldness score: F_1_ = 0.788, *P* = 0.379; hesitancy: F_1_ = 0.035, *P* = 0.853) and thus excluded from any further analysis.

To compare the strength of individual relationships, all regression coefficients (*β*) were standardized by multiplying with the phenotypic standard deviation of the predictor variable and dividing by the phenotypic standard deviation of the response variable (Schielzeth 2010). In the case of non-Gaussian distributed responses, the phenotypic standard deviation of the response variable was indirectly estimated according to Menard (2011). The resulting effect sizes (β^*^) take values between − 1 and 1.

## Results

### Environmental sources of variation in black spot infestation among populations

**Fig. 1:**
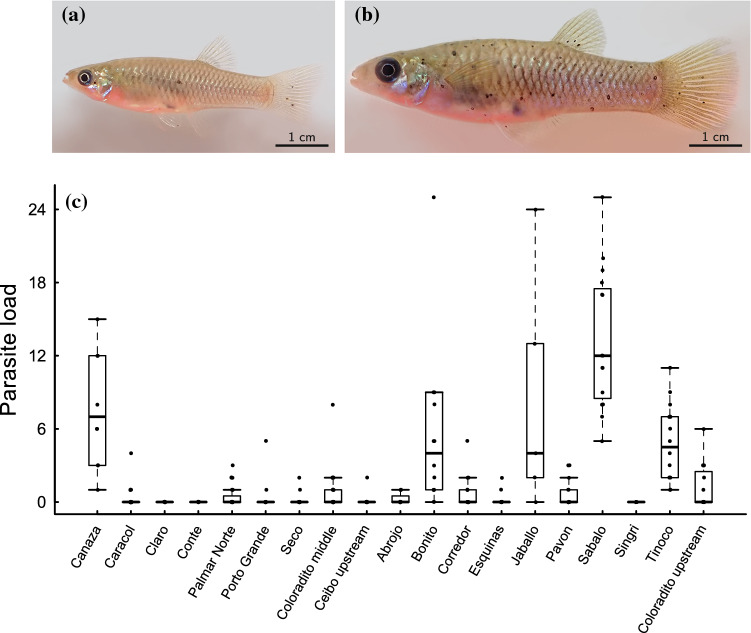
**a** Immature and **b** adult female *Poeciliopis retropinna* with low and high parasite load (number of black spots), respectively. **c** Boxplot showing variation in parasite load among and within sampling locations (i.e. populations)

**Fig. 2:**
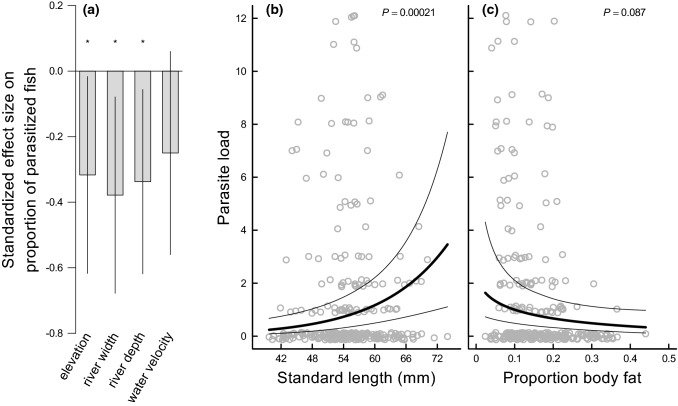
**a** Standardized effect size (± 95% CI) of the environmental variables (elevation of the sampling location, mean river width, mean river depth, mean water velocity) on the proportion of parasitized *Poeciliopsis retropinna* females within a sampling population. The effect sizes are calculated based on the model-averaged parameters given in Online Resource Table S4 and take values between − 1 and 1. Significant relationships are indicated with an asterisk (^*^). **b** Individual parasite load (number of black spots per female) as a function of maternal standard length (*n* = 302). **c** Individual parasite load as a function of proportion of maternal body fat (*n* = 302). The relationships in **b** and **c** were estimated in the generalized linear mixed effects model described in Online Resource Table S5. The model predictions account for the proportion of maternal body fat in **b** and maternal standard length in **c** that are kept constant at the overall mean (i.e. body fat = 0.15, standard length = 55 mm). Data points correspond to the ‘jittered’ raw data. *P*-value is given at the top

We found substantial variation in black spot infestation among and within the populations of *Poeciliopsis retropinna* (Fig. 1). Particularly, the proportion of parasitized individuals was negatively correlated with elevation of the sampling location (β^*^ = − 0.317, *z* = 2.068, *P* = 0.039; Online Resource Table S4; Fig. 2a), mean river width (β^*^ = − 0.378, *z* = 2.475, *P* = 0.013; Online Resource Table S4; Fig. 2a), and mean river depth (β^*^ = − 0.337, *z* = 2.351, *P* = 0.019; Online Resource Table S4; Fig. 2a). Moreover, the proportion of parasitized individuals tended to negatively correlate with mean water velocity (β^*^ = − 0.250, *z* = 1.580, *P* = 0.114; Online Resource Table S4; Fig. 2a).

### Maternal sources of variation in black spot infestation within populations

Individual parasite load (number of black spots per female) was positively associated with maternal standard length (β^*^ = 0.302, *z* = 3.708, *P* < 0.001; Online Resource Table S5; Fig. 2b) and tended to decrease with increasing proportion of maternal body fat, though not significantly (β^*^ = − 0.186, *z* = − 1.709, *P* = 0.087; Online Resource Table S5; Fig. 2c).

### Life-history consequences of black spot infestation

**Fig. 3:**
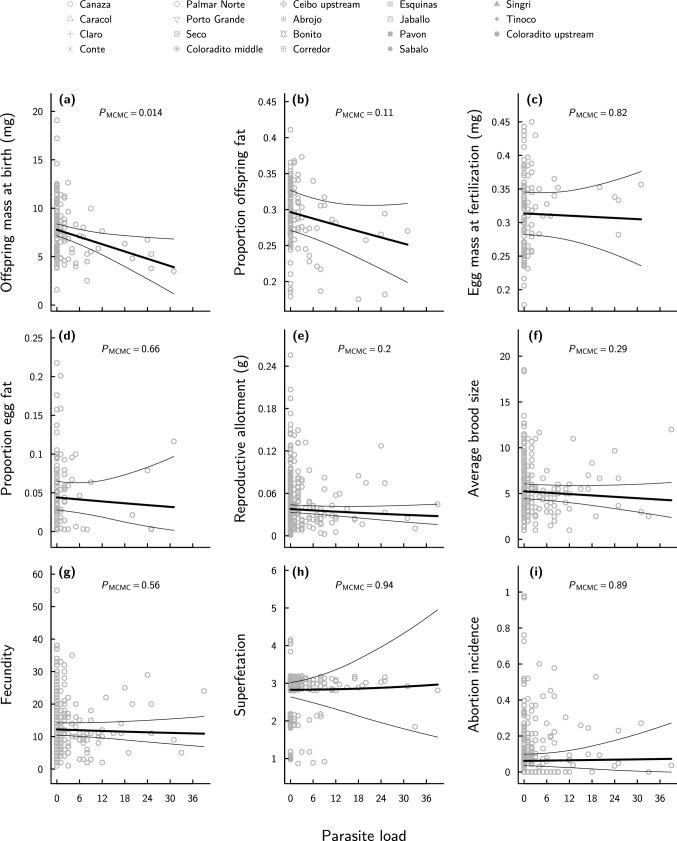
Life-history characteristics of *Poeciliopsis retropinna* in relation to black spot infestation. **a** Offspring dry mass at birth (developmental stage 45) (*n* = 132), **b** proportion offspring fat at birth (*n* = 132), **c** egg dry mass at fertilization (developmental stage 2) (*n* = 109), **d** proportion egg fat at fertilization (*n* = 99), **e** absolute dry reproductive allotment (*n* = 287), **f** average brood size (*n* = 282), **g** maternal fecundity (number embryos in all broods combined) (*n* = 282), **h** degree of superfetation (*n* = 288), and **i** abortion incidence (*n* = 288) (± 95% CI) as a function of parasite load (number of black spots per female) estimated in the models described in Online Resource Table S6. All model predictions account for the proportion of maternal body fat and maternal standard length, which are kept constant at the overall population mean (i.e. body fat = 0.15, standard length = 55 mm). In **e** and **g**–**h**, the developmental stage of the most-developed brood carried by the female is kept constant at the overall median (i.e. developmental stage 42.5). Data points correspond to the population-specific raw data. *P*_MCMC_-value is given at the top of each panel

**Fig. 4:**
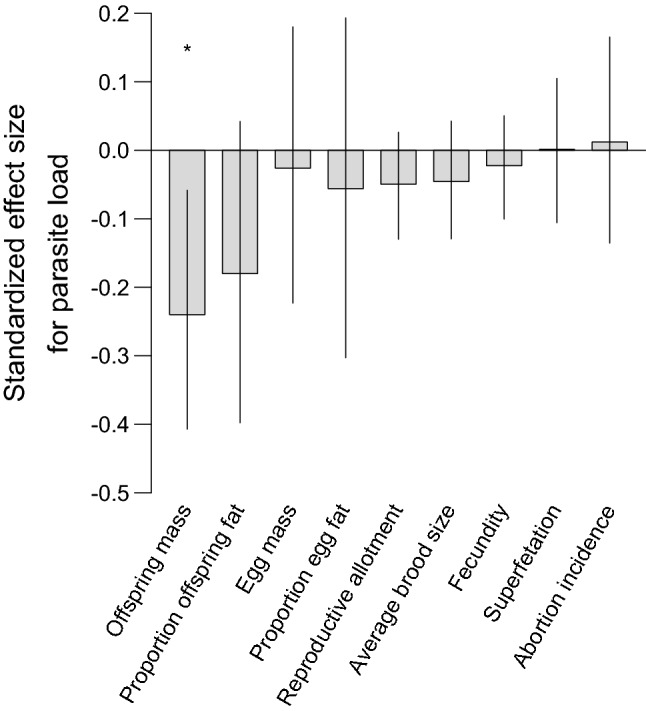
Standardized effect size (± 95% CI) of parasite load (number of black spots per female) on maternal life-history traits (offspring dry mass at birth, proportion offspring fat at birth, egg dry mass at fertilization, proportion egg fat at fertilization, absolute dry reproductive allotment, average brood size, fecundity, superfetation, abortion incidence). The effect sizes take values between − 1 and 1. Significant relationships are indicated with an asterisk (^*^)

Independent of maternal standard length and body fat, heavily parasitized female *P. retropinna* produced smaller offspring at birth (β^*^_post.mean_ = − 0.240, 95% CI − 0.407– − 0.058, *P*_MCMC_ = 0.014; Online Resource Table S6a; Figs. 3a, 4) that tended to have a lower proportion of body fat (β^*^_post.mean_ = − 0.180, 95% CI − 0.398*–*0.042, *P*_MCMC_ = 0.110; Online Resource Table S6b; Figs. 3b, 4). In contrast, parasite load did not correlate with egg mass (β^*^_post.mean_ = − 0.026, 95% CI − 0.223–0.180, *P*_MCMC_ = 0.818; Online Resource Table S6c; Figs. 3c, 4) or the proportion of egg fat at fertilization (β^*^_post.mean_ = − 0.056, 95% CI − 0.303–0.193, *P*_MCMC_ = 0.658; Online Resource Table S6d; Figs. 3d, 4). Likewise, parasite load was not associated with absolute dry reproductive allotment (β^*^_post.mean_ = − 0.050, 95% CI − 0.130–0.027, *P*_MCMC_ = 0.198; Online Resource Table S6e; Figs. 3e, 4), average brood size (β^*^_post.mean_ = − 0.046, 95% CI − 0.129*–*0.043, *P*_MCMC_ = 0.288; Online Resource Table S6f; Figs. 3f, 4), fecundity (β^*^_post.mean_ = − 0.022, 95% CI − 0.101–0.051, *P*_MCMC_ = 0.560; Online Resource Table S6g; Figs. 3g, 4), superfetation (β^*^_post.mean_ = 0.001, 95% CI − 0.106–0.105, *P*_MCMC_ = 0.936; Online Resource Table S6h; Figs. 3h, 4), or abortion incidence (β^*^_post.mean_ = 0.012, 95% CI − 0.136–0.165, *P*_MCMC_ = 0.892; Online Resource Table S6i; Figs. 3i, 4).

### Boldness responses to black spot infestation

**Fig. 5:**
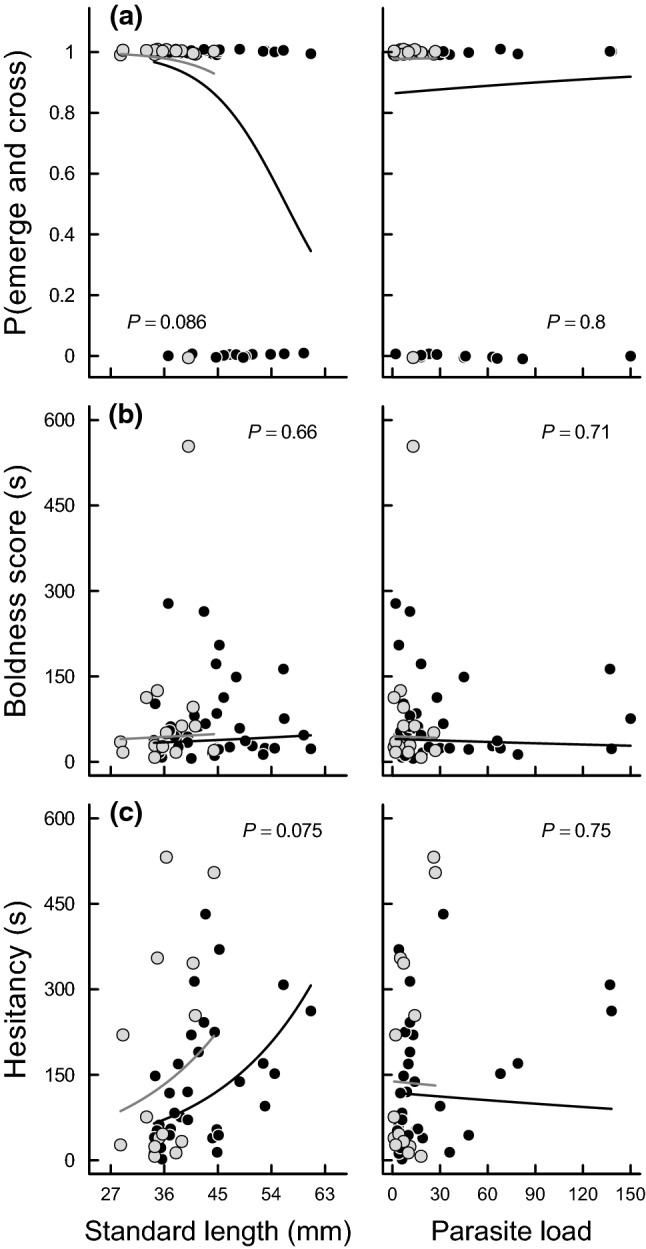
Boldness responses of *Poeciliopsis retropinna* to black spot infestation assessed in the field. **a** Probability of emerging from the box and crossing the metal ring (*n* = 60), **b** boldness score (time taken to emerge from the box) (*n* = 56), and **c** hesitancy (the time the fish took to cross the metal ring minus the time it took to emerge from the box) (*n* = 46) as a function of sex (black line: females; grey line: males), standard length (left panels) and parasite load (number of black spots; right panels) estimated in the models described in Online Resource Table S7–S9. All model predictions account for the number of parasites (left panels) and standard length (right panels), which are kept constant at the sex-specific population mean (i.e. number parasites_♀_ = 29, number parasites_♂_ = 10, standard length_♀_ = 44, standard length_♂_ = 36 mm). Measurement day is kept constant at day 1. Data points (black: females; grey: males) correspond to the raw data; ‘jittered’ in **a**. *P* value for the effect of standard length (left panels) and parasite load (right panels) are given in each panel

The probability of emerging from the box and crossing the metal ring tended to decrease with increasing standard length, though not significantly (β^*^ = − 0.732, *z* = − 1.715, *P* = 0.086; Online Resource Table S7; Fig. 5a). This decrease was similar in females and males (β^*^ = 0.228, *z* = 0.663, *P* = 0.507; Online Resource Table S7; Fig. 5a). In contrast, the probability of emerging from the box and crossing the metal ring did not correlate with parasite load (number of black spots) (β^*^ = 0.084, *z* = 0.254, *P* = 0.800; Online Resource Table S7; Fig. 5a), but tended to correlate with measurement day (β^*^ = − 0.443, *z* = − 1.761, *P* = 0.078; Online Resource Table S7).

The boldness score (the time taken to emerge from the box) did not correlate with standard length (β^*^ = 0.082, *t* = 0.448, *P* = 0.656; Online Resource Table S8; Fig. 5b), sex (β^*^ = 0.080, *t* = 0.606, *P* = 0.547; Online Resource Table S8; Fig. 5b), and parasite load (β^*^ = − 0.070, *t* = − 0.372, *P* = 0.712; Online Resource Table S8; Fig. 5b). However, fish measured on day 2 took longer to emerge from the box than fish measured on day 1 (β^*^ = 0.435, *t* = 3.232, *P* = 0.002; Online Resource Table S8).

Finally, hesitancy (the time the fish took to cross the metal ring minus the time it took to emerge from the box) tended to increase with increasing standard length, though not significantly (β^*^ = 0.375, *t* = 1.824, *P* = 0.075; Online Resource Table S9; Fig. 5c). Moreover, males tended to be more hesitant than females (β^*^ = 0.249, *t* = 1.853, *P* = 0.071; Online Resource Table S9; Fig. 5c). In contrast, hesitancy was not associated with parasite load (β^*^ = − 0.055, *t* = − 0.325, *P* = 0.747; Online Resource Table S9; Fig. 5c) or measurement day (β^*^ = 0.198, *t* = 1.650, *P* = 0.107; Online Resource Table S9).

## Discussion

Parasites can affect the survival and reproductive success of their hosts (Bush et al. 2001). In order to reduce these fitness costs of parasitism, hosts can respond on a physiological, morphological or behavioral level (Richner 1998). Here we examined the importance of black spot disease (BSD) in shaping life history and behavior in the placental live-bearing fish species *Poeciliopsis retropinna*. We found substantial variation in black spot infestation among and within the study populations. The proportion of parasitized individuals in a population was negatively correlated with elevation of the sampling location, mean river width, mean river depth, and tended to negatively correlate with mean water velocity. Within populations, individual parasite load (i.e. the number of black spots per female) was positively associated with maternal standard length, but tended to decrease with increasing proportion of maternal body fat. Parasite load did not correlate with other maternal life-history traits, such as fecundity, reproductive allotment, superfetation, or abortion incidence. However, heavily parasitized females produced smaller offspring at birth that tended to have a smaller proportion of body fat, while the size and quality of eggs at fertilization remained unaffected. Finally, independently of body size, parasite load was not associated with an individual’s boldness (boldness score and hesitancy).

### Environmental sources of variation in black spot infestation among populations

The trematode that is causing BSD relies on water snails and fish as intermediate, and piscivorous birds as final, hosts during its life-cycle (Lane and Morris 2010). Environmental variables are thereby likely to affect the relative abundance of hosts and the probability of free-living parasite life stages successfully invading the hosts. Relating parasitism to environmental variables may, therefore, shed light on the abiotic conditions that favor the chance of the parasite completing its life-cycle.

For example, we found that the proportion of parasitized individuals increases with decreasing elevation of the sampling location. In other words, the probability of BSD is higher in coastal rivers near the ocean compared to high-altitude inland rivers. In Costa Rica, inland rivers are typically enclosed by dense canopy and drain from steep mountain environments, whereas lowland rivers are often free of vegetation. Piscivorous birds are visually oriented predators and shade due to increased vegetation cover decreases their foraging success (Trexler et al. 1994; Penaluna et al. 2016). Bird predation is thus likely higher for fish in the open canopy lowland rivers compared to the closed canopy inland rivers. Because piscivorous birds are obligatory final hosts, open canopy areas may increase the chance of the parasite completing its life-cycle, thereby partly explaining the higher proportion of infested females in lowland regions. A decrease in elevation is furthermore associated with an increase in water temperature. The production of cercariae in intermediate snail hosts has been shown to be profoundly influenced by increasing temperature, which causes an increase in cercarial output and hence a greater number of cercarial infective stages in aquatic habitats (Poulin 2006). Thus, the increasing proportion of infected individuals with decreasing elevation may be the result of both warmer water and open canopies, which facilitate parasite reproduction in their intermediate and final hosts, respectively.

We further found that the proportion of parasitized individuals decreases with increasing river width and depth. In other words, the probability of BSD is higher in smaller (i.e. narrower and shallower) compared to larger (i.e. wider and deeper) rivers. The density of the parasite’s intermediate hosts (i.e. the number of water snails and fish per unit volume of water) is likely to be inversely related to river size. As a result, the chance of the parasite finding a host may decrease with increasing river size. Moreover, piscivorous birds are more effective predators in shallow near-shore water (Whitfield and Cyrus 1978; Kramer et al. 1983). This means that the higher observed proportion of infested individuals with decreasing river with and depth might be related to both a higher parasite density and higher probability of parasitized fish getting eaten by their final hosts thereby completing the parasite life-cycle.

The probability of BSD further tended to decrease with increasing water velocity, being higher in slow flowing or stagnant rivers compared to fast flowing rivers. This may be related to the locomotor ability of the miracidia and cercariae that infect water snails and fish, respectively. Both of these life stages are relatively small (< 1 mm) and although they are motile free-living stages (Hoffman and Putz 1965), they have very limited swimming capabilities (Koehler et al. 2012). The chance that free-swimming miracidia and cercariae find and successfully invade intermediate snail or fish hosts might therefore decrease with increasing flow velocities, because they may get washed away before successfully invading a host (Marcogliese 2016).

Nevertheless, the observed correlations with parasite load should be interpreted with care, as we only measured a limited set of environmental variables. The exclusion of variables that are potentially important for parasite ecology could lead to spurious correlations. Therefore, future studies focusing on parasite ecology should include a broader set of local environmental variables (e.g. density of snails and piscivorous birds).

### Maternal sources of variation in black spot infestation within populations

We found that individual parasite load (number of black spots per female) is positively correlated with maternal standard length: larger fish carry more parasites. This is likely due to two non-mutually exclusive effects: a time (age) effect and a fish size effect. First, the fibrous capsule and melanocyst wall surrounding the parasite prevent parasites from easily being exocytosed from the host (Cureton et al. 2011). This means that parasites will remain embedded in the skin until the fish dies (or gets eaten by the parasite’s final host) and that the number of parasites on an individual will (independent of fish size) increase over time due to continued exposure to the parasites. Second, larger fish are simply able to carry more parasites, because growth is associated with an increase in skin surface area to which parasites can attach. Therefore, the observed relationship between parasite infestation and standard length may be the result of (1) an accumulation of parasite infection over time (with age), and (2) increase in skin surface area to which parasites can attach during body growth.

Independent of standard length, parasite load tended to be higher for females with low proportion of body fat, though not significantly. The proportion of maternal body fat is believed to be a good indicator of fish condition (Leips et al. 2013) and may thus affect, or be affected by, parasitism. Specifically, a poor body condition might be expected to be associated with a high parasite load, either because worse-conditioned individuals are more susceptible to the parasite (less energy for immune function), or the host’s response to the parasite encapsulation is energetically expensive (Lane and Morris 2010; Cureton et al. 2011). For instance, BSD was shown to reduce the body condition of smallmouth bass (*Micropterus dolomieui*) (Hunter and Hunter 1938) and juvenile bluegill sunfish (*Lepomis macrochiris*) (Lemly and Esch 1984). However, there was no evidence for a reduced body condition in infected females of the poeciliid fish *Gambusia affinis* (Cureton et al. 2011). Likewise, there was no clear association of parasite load with body condition in our study, and thus, BSD may not invoke an energetic demand strong enough to reduce maternal fat reserves in *P. retropinna*.

### Life-history consequences of black spot infestation

Parasites can change the allocation of resources to different functions such as growth, reproduction, survival, and maintenance by inducing shifts in the optimum of life-history traits (Michalakis and Hochberg 1994; Sheldon and Verhulst 1996; Richner 1998).

We found that BSD does not correlate with maternal fecundity, reproductive allotment, superfetation, or abortion incidence. However, independent of maternal body fat and size, heavily parasitized females produce smaller and worse-conditioned offspring at birth, while egg size and quality at fertilization remain unaffected. Specifically, an increase in maternal parasite load by five black spots, decreases the mass of offspring at birth by 8.73% and the proportion of offspring body fat by 2.57%. In placental live-bearing fish, resource allocation to offspring takes place during two distinct periods (Wourms 1981). Part of the nutrients are supplied pre-fertilization during oogenesis and stored as high-energy yolk (Wallace and Selman 1981). The rest of the nutrients are transferred post-fertilization to the developing embryos throughout gestation (Wourms 1981). The latter is achieved through a follicular placenta, roughly an analog to the mammalian placenta (Turner 1940). In general, the amount of resources a female can transfer to her developing offspring per unit of time is the result of a balance between maternal energy uptake (via feeding), her own caloric utilization (maintenance) and the amount of excess energy that is subsequently available for reproduction (Stearns 1992). In parasitic environments, hosts may allocate an increased amount of resources to parasite defense that might otherwise have been used for different functions (Sheldon and Verhulst 1996). It is thus possible that parasitized females simply have less energy available that can be used to invest in developing embryos, which results in the production of smaller and worse-conditioned offspring at birth.

Furthermore, the degree of post-fertilization maternal provisioning has been shown to be positively correlated with the elaboration of the structures that form the interface between the mother and offspring, and hence to reflect the complexity of placental morphology (Kwan et al. 2015; Olivera-Tlahuel et al. 2018). Producing such a highly elaborate tissue is presumably energetically costly. In mice, maternal malnutrition was shown to impact placental morphology, which consequently leads to impaired placental functioning and insufficient nutrient provisioning to embryos (Connor et al. 2020). In placental poeciliid fish, maternal malnutrition is known to lead to smaller offspring at birth (Reznick et al. 1996; Banet et al. 2010; Pollux and Reznick 2011). Smaller offspring have lower competitive abilities (Bashey 2006) and lower swimming capabilities (e.g. Dial et al. 2016; Lankheet et al. 2016), and thus, presumably lower fitness in adverse resource environments (Reznick et al. 1996; Banet et al. 2010; Pollux and Reznick 2011). However, to what extent parasitism might potentially negatively impact placental functioning in poeciliid fish, either directly or indirectly by shifting the energy investment required to form an elaborate placental structure more towards other functions, is currently unknown and requires further investigation.

### Boldness responses to black spot infestation

Parasitism plays a potentially important role in shaping aspects of animal personalities, such as exploration, activity or boldness (Barber and Dingemanse 2010). We found that parasite load does not correlate with our measurement of boldness (boldness score and hesitancy) in *P. retropinna*, suggesting that potential parasite-induced behavioral modifications are not reflected by an individual’s boldness in this species. Bolder individuals typically engage in more risky behavior to obtain food (Chapman et al. 2010). This increases resource-intake rates, but also their vulnerability to predators (Godin and Smith 1988). The adaptive significance of risk taking, therefore, depends on the cost/benefit ratio of being bold in different situations (Sih et al. 2004). Specifically, parasitism may favor high levels of boldness, when the compensation of the parasite-induced fitness costs is efficient (Kortet et al. 2010). For instance, increased boldness may enable parasitized individuals to effectively compensate for the extra nutritional requirements caused by the parasite (Giles 1983). Alternatively, parasitism may favor low levels of boldness, when individuals have little or no means of compensating for the costs (Kortet et al. 2010). In *P. retropinna*, therefore, BSD may not invoke fitness costs large enough to favor either increased or decreased levels of boldness. This is in line with our findings showing that BSD is only weakly associated with a reduced maternal body condition (body fat reserves) or fecundity.

In contrast, we found that the probability of emerging from the box and crossing the metal ring tends to decrease with increasing standard length in males and females. Moreover, if an individual emerges from the box and crosses the metal ring, larger fish take longer to do so. In other words, larger fish tend to be more hesitant. However, although the effect sizes for both relationships were moderate to large (β^*^_probability_ = −0.732; β^*^_hesitancy_ = 0.375) neither were significant, which is likely the result of the small sample size and hence low statistical power. The negative relationship between boldness and body size was best explained by the so-called metabolic hypothesis in other poeciliids (Brown and Braithwaite 2004; Brown et al. 2005). Specifically, smaller individuals have lower body fat reserves and faster metabolic rates, and may thus be compelled to leave a secure shelter sooner to maximize feeding opportunities (Krause et al. 1998; Skalski and Gilliam 2002).

## Conclusion

Parasites can change the allocation of resources to different functions such as growth, reproduction, survival, and maintenance by inducing shifts in the optimum of life-history traits (Michalakis and Hochberg 1994; Sheldon and Verhulst 1996; Richner 1998). Placental live-bearing fish transfer nutrients to their developing embryos throughout pregnancy via a placenta (Wourms 1981). Parasite infestation of females during pregnancy could potentially influence embryo development at different gestational stages. We show that BSD is associated with a reduction in post-fertilization maternal provisioning resulting in the production of smaller and worse-conditioned offspring at birth. These parasitized females may be physiologically unable to produce larger offspring, either because of less resources available to invest in developing embryos or because of placental malfunctioning. Our study herewith identified an important potential cost of trematode infestation in placental live-bearing fish, which is a reduced maternal nutrient provisioning to embryos, resulting in smaller size and quality, and potentially lower fitness, of offspring at birth.

## Electronic supplementary material

Below is the link to the electronic supplementary material.

Supplementary file1 (PDF 297 KB)

## Data Availability

The data that support the findings of this study are available from Dryad Digital Repository: 10.5061/dryad.6hdr7sqxr.
