# Supplementary material for: Parasite infestation influences life history but not boldness behavior in placental live-bearing fish
Source: Oecologia. 2020 Nov 3;194(4):635–48. doi: 10.1007/s00442-020-04795-6 (PMC7683485; doi:10.1007/s00442-020-04795-6)
Supplement: Supplementary file 1 — Supplementary file1 (PDF 297 KB) [file 442_2020_4795_MOESM1_ESM.pdf]

Electronic Supplementary Materials for

Parasite infestation influences life history but not boldness behavior in placental live-bearing fish

Andres Hagmayer[1], **Andrew I. Furness**[2,3], **and Bart J. A. Pollux**[1,*]

[1] Experimental Zoology Group, Department of Animal Sciences, Wageningen University, 6708 WD Wageningen, Netherlands
[2] Department of Ecology and Evolutionary Biology, University of California, CA 92697 Irvine, USA
[3] Department of Biological and Marine Sciences, University of Hull, HU6 7RX Hull, UK

[*] **Corresponding author:**
E-mail: bart.pollux@wur.nl
Phone number: +31.(0)317.486083
Fax number: n/a

1 Supplementary Methods

1.1 Measurements of water parameters

The water velocity was measured at each location to the nearest 0.01 $m{\cdot}s^{-1}$ with a Höntzsch Vane Wheel FA current meter (type ZS30 GFE md20 T/100-2/p10, Höntzsch Instruments, Waiblingen, Germany). Depending on the uniformity of the flow, the water velocity was taken at 9–17 incremental observation points across a transect of the stream. At each observation point, the mean water velocity was defined as the average of three repeated measurements at a height above the stream bed equal to 0.4 times the depth at that location. When the water depth exceeded 60 cm, the mean water velocity was calculated as the average between the velocities measured at 0.2 times the water depth and 0.8 times the water depth [4].

1.2 Laboratory measurements

The standard length of preserved specimens was measured to the nearest mm from the tip of the upper jaw to the outer margin of the hypural plate, using a caliper. Female dry mass was measured to the nearest 0.01 mg on a Mettler Toledo XP205 delta range (Mettler Instruments Corp., Hightstown, New Jersey, USA) after removing the ovary and air-drying the female overnight at 60°C in a drying oven. Female lean mass was measured by extracting the fat twice with anhydrous diethyl ether (Fisher Scientific) to remove triglycerides, and by subsequently air-drying and re-weighting the female (see above). The proportion of maternal body fat was then calculated by subtracting maternal lean mass from maternal dry mass divided by maternal dry mass. The embryo dry mass for a given brood was calculated as the dry mass of the brood, measured to the nearest 0.01 mg after air-drying overnight at 60°C (see above), divided by the number of embryos in the brood [5].

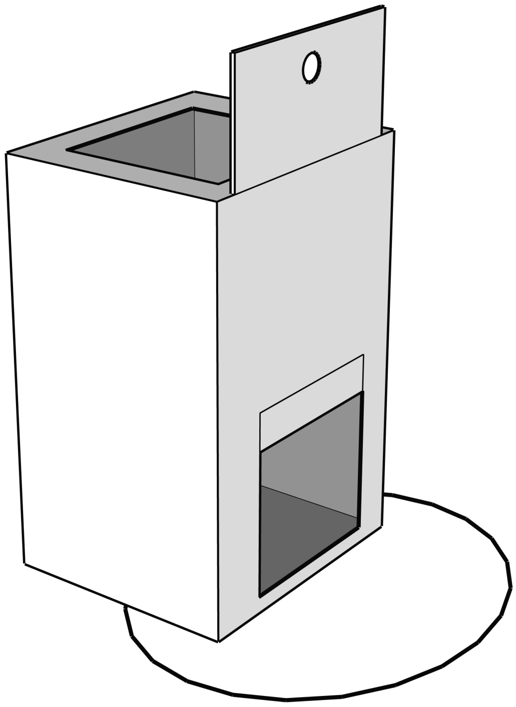

Fig. S1. Schematic illustration of the plastic box (17×12×11.5 cm) used to assess an individual's boldness in the field. The box was positioned in approximately 15 cm of water on the edge of the pool. A metal ring was placed underneath the box to form a D in a radius of 8 cm in front of the box. A single fish was placed at a time in the box with a lid on the top. After an acclimation period of 2 min, a trapdoor (7.5 cm wide and 9.5 cm high) in the front of the box was opened and the fish was free to emerge and to swim into the pool. Individual fish were observed from above until they crossed the metal ring.

Table S1. Sampling locations (plus coordinates), sampling dates, parasitism (i.e. proportion of parasitized females), and number of collected (*n*) and pregnant (n_{preg}) females at each location.

Location	River	Parasitism	Coordinates	Sampling date	*n*	n_{preg}
Canaza	Rio Canaza	1.00	N 08° 38', W 83° 10'	08.03.2017	10	10
Caracol	Rio Caracol	0.16	N 08° 39', W 83° 00'	09.03.2017	25	25
Claro	Rio Coto Colorado	0.00	N 08° 41', W 83° 06'	09.03.2017	15	15
Conte	Rio Conte	0.00	N 08° 26', W 83° 02'	07.03.2017	7	7
Palmar Norte	Rio Balsar	0.25	N 08° 59', W 83° 31'	11.03.2017	32	31
Seco	Rio Seco	0.08	N 08° 39', W 82° 56'	06.03.2017	25	23
Coloradito middle	Rio Coloradito	0.27	N 08° 35', W 82° 52'	05.03.2017	37	36
Porto Grande	Rio Guineal	0.20	N 09° 01', W 83° 09'	01.03.2017	10	10
Ceibo upstream	Rio Ceibo	0.05	N 09° 12', W 83° 18'	27.02.2017	22	21
Bonito	Rio Bonito	0.87	N 08° 43', W 83° 12'	25.03.2018	15	14
Esquinas	Rio Esquinas	0.10	N 08° 44', W 83° 10'	24.03.2018	20	17
Sabalo	Rio Sabalo	1.00	N 08° 52', W 83° 19'	26.03.2018	15	15
Abrojo	Rio Abrojo	0.25	N 08° 37', W 82° 52'	23.03.2018	12	12
Corredor	Rio Corredor	0.30	N 08° 40', W 82° 54'	23.03.2018	20	20
Jaballo	Quebrada Jaballo	0.80	N 08° 57', W 83° 06'	16.03.2018	5	5
Pavon	Rio Balsar	0.29	N 09° 00', W 83° 31'	26.03.2018	17	17
Singri	Rio Singri	0.00	N 08° 59', W 83° 06'	15.03.2018	6	6
Tinoco	Rio Tinoco	1.00	N 08° 54', W 83° 22'	26.03.2018	18	18
Coloradito upstream	Rio Coloradito	0.35	N 08° 35', W 82° 51'	25.03.2018	20	20
Total					331	322

Table S2. **Parameter estimates of the generalized linear models predicting variation in the probability of black spot infestation. The probability of black spot infestation was modeled as the proportion of parasitized individuals per sampling location using maximum likelihood and a logit link for the binomial-distributed response.**

Model	Int	elevation	river depth	river width	water velocity	*df*	logLik	AICc	$\triangle$AICc	weight
1	-0.825	-0.470		-0.546		3	-92.301	192.203	0.000	0.227
2	-0.794		-0.506		-0.401	3	-92.804	193.208	1.005	0.137
3	-0.849	-0.389	-0.480			3	-92.817	193.234	1.031	0.136
4	-0.870	-0.311	-0.471		-0.321	4	-91.211	193.279	1.076	0.133
5	-0.848	-0.434		-0.513	-0.229	4	-91.469	193.796	1.593	0.102
6	-0.840	-0.428	-0.246	-0.345		4	-91.512	193.880	1.677	0.098
7	-0.864	-0.370	-0.285	-0.274	-0.265	5	-90.457	195.530	3.327	0.043
8	-0.777		-0.396	-0.161	-0.365	4	-92.520	195.897	3.694	0.036
9	-0.750		-0.543			2	-95.837	196.425	4.222	0.028
10	-0.720			-0.479	-0.311	3	-94.549	196.699	4.496	0.024
11	-0.726		-0.340	-0.285		3	-94.837	197.274	5.071	0.018
12	-0.685			-0.550		2	-96.381	197.513	5.310	0.016
13	-0.832	-0.376			-0.336	3	-97.736	203.072	10.870	0.001
14	-0.818	-0.468				2	-99.760	204.269	12.067	0.001
15	-0.740				-0.449	2	-100.481	205.713	13.510	0.000
16	-0.698					1	-105.112	212.460	20.257	0.000

Int: intercept; elevation: z-standardized elevation of sampling location (m.a.s.l.); river depth: z-standardized mean river depth (m); river width: z-standardized mean river width (m); velocity: z-standardized mean water velocity ($m \cdot s^{-1}$); AICc: Akaike's information criterion adjusted for small sample sizes.

Table S3. Pairwise Pearson correlation between the measured environmental variables at each sampling location.

	water velocity	river depth	river width	elevation
water velocity	1.000	0.217	0.298	0.410
river depth	0.217	1.000	0.821	0.225
river width	0.298	0.821	1.000	0.163
elevation	0.410	0.225	0.163	1.000

Table S4. **Model-averaged parameters predicting variation in the probability of black spot infestation. The probability of black spot infestation was modeled as the proportion of parasitized individuals per sampling location using maximum likelihood and a logit link for the binomial-distributed response. Particularly, each parameter estimate is averaged over the models in the 'top model set' ($\triangle \mathbf{AICc} < 2$; Table S2) in which that predictor appears and is weighted by the summed weights of these models [2].**

	Estimates					
	β	SE	adj. SE	*z*	*P*	Sig.
intercept	-0.835	0.139	0.150	5.566	0.000	***
elevation*	-0.413	0.185	0.199	2.068	0.039	*
river width*	-0.492	0.186	0.199	2.475	0.013	*
river depth*	-0.439	0.176	0.187	2.351	0.019	*
water velocity*	-0.325	0.192	0.206	1.580	0.114	n.s.

**z*-standardized; β: regression coefficient; significant codes: $P < 0.001^{***}$, $< 0.01^{**}$, $\leq 0.05^{*}$, > 0.05 n.s.

Table S5. Fixed and random effects in explaining variation in parasite load (number of black spots per female) estimated in a generalized linear mixed model using maximum likelihood and a log link for the negative binomial-distributed response. The model was fitted using the package `glmmTMB` [1].

Fixed effects					
	β	SE	*z*	*P*	Sig.
intercept	-3.339	1.584	-2.109	0.035	*
proportion maternal body fat*	-3.041	1.780	-1.709	0.087	n.s.
maternal standard length	0.078	0.021	3.708	0.000	***

Random effects			
	Variance	2.5% confidence level	97.5% confidence level
population	0.1478	0.0004	0.6588

*arcsin square-root transformed; β: regression coefficient; significant codes: $P < 0.001^{***}$, $< 0.01^{**}$, $\leq 0.05^{*}$, > 0.05 n.s.

Table S6. **Fixed and random effects in explaining variation in maternal life history. (a) Offspring dry mass at birth (developmental stage 45), (b) proportion offspring fat at birth, (c) egg dry mass at fertilization (developmental stage 2), (d) proportion egg fat at fertilization, (e) absolute dry reproductive allotment, (f) average brood size, (g) maternal fecundity (number embryos in all broods combined), (h) degree of superfetation, and (i) abortion incidence estimated using the `MCMCglmm` package [3]. All traits were fitted in a multivariate (generalized) linear mixed effects model allowing for the covariance between the residuals of all responses. The default normal priors were used for the fixed effects with the expected value of 0 and variance 10^{12}. Inverse-Wishart priors were used for the residual variances with the expected value of 1 and degree of belief of 0.02. The number of iterations was 5,500,000, with a burnin of 500,000 and a thinning of 5000.**

(a) Offspring dry mass at birth

Fixed effects

	$\beta_{\text{post.mean}}$	2.5% CI	97.5% CI	eff.samp	P_{MCMC}	Sig.
intercept	0.717	-4.129	5.755	1181.800	0.754	n.s.
parasite load*	-0.125	-0.212	-0.029	1073.222	0.014	*
maternal standard length	0.062	-0.012	0.137	1000.000	0.100	n.s.
proportion maternal body fat†	9.392	3.697	15.395	1000.000	0.001	***

Random effects

	$\sigma^2_{\text{post.mean}}$	2.5% CI	97.5% CI	eff.samp
population identity	0.6397	0.0048	1.8228	1000.0000

(b) Proportion offspring fat at birth

Fixed effects

	$\beta_{\text{post.mean}}$	2.5% CI	97.5% CI	eff.samp	P_{MCMC}	Sig.
intercept	0.398	0.291	0.495	1129.907	0.001	***
parasite load*	-0.001	-0.003	0.000	1000.000	0.110	n.s.
maternal standard length	0.001	-0.001	0.002	1000.000	0.216	n.s.
proportion maternal body fat†	0.263	0.136	0.365	1000.000	0.001	***

Random effects

	$\sigma^2_{\text{post.mean}}$	2.5% CI	97.5% CI	eff.samp
population identity	0.0026	0.0011	0.0050	1000.0000

(c) Egg dry mass at fertilization

Fixed effects

	$\beta_{\text{post.mean}}$	2.5% CI	97.5% CI	eff.samp	P_{MCMC}	Sig.
intercept	0.154	0.037	0.279	1000.000	0.014	*
parasite load*	-0.000	-0.003	0.002	612.852	0.818	n.s.
maternal standard length	0.003	0.002	0.005	1000.000	0.001	***
proportion maternal body fat†	-0.026	-0.182	0.103	1000.000	0.722	n.s.

Random effects

	$\sigma^2_{\text{post.mean}}$	2.5% CI	97.5% CI	eff.samp
population identity	0.0037	0.0016	0.0069	1000.0000

(d) Proportion egg fat at fertilization						
Fixed effects						
	$\beta_{\text{post.mean}}$	2.5% CI	97.5% CI	eff.samp	P_{MCMC}	Sig.
intercept	-0.028	-0.346	0.228	1000.000	0.852	n.s.
parasite load*	-0.001	-0.005	0.004	1201.242	0.658	n.s.
maternal standard length	0.003	-0.001	0.007	1000.000	0.248	n.s.
proportion maternal body fat†	0.244	-0.090	0.535	1000.000	0.124	n.s.

Random effects				
	$\sigma^2_{\text{post.mean}}$	2.5% CI	97.5% CI	eff.samp
population identity	0.0047	0.0013	0.0093	1000.0000

(e) Absolute dry reproductive allotment						
Fixed effects						
	$\beta_{\text{post.mean}}$	2.5% CI	97.5% CI	eff.samp	P_{MCMC}	Sig.
intercept	-7.020	-7.812	-6.239	1000.000	0.001	***
parasite load*	-0.009	-0.021	0.006	1000.000	0.198	n.s.
maternal standard length	0.069	0.057	0.080	1000.000	0.001	***
proportion maternal body fat†	-0.164	-0.988	0.719	1000.000	0.722	n.s.
latest stage‡	0.091	0.078	0.103	887.257	0.001	***
latest stage^{2}§	0.141	0.101	0.180	1000.000	0.001	***
parasite load*×latest stage‡	-0.000	-0.001	0.001	1000.000	0.648	n.s.

Random effects				
	$\sigma^2_{\text{post.mean}}$	2.5% CI	97.5% CI	eff.samp
population identity	0.0806	0.0234	0.1661	909.5209

(f) Average brood size						
Fixed effects						
	$\beta_{\text{post.mean}}$	2.5% CI	97.5% CI	eff.samp	P_{MCMC}	Sig.
intercept	-10.298	-13.282	-7.187	1000.000	0.001	***
parasite load*	-0.025	-0.073	0.021	1101.574	0.288	n.s.
maternal standard length	0.316	0.275	0.359	1000.000	0.001	***
proportion maternal body fat†	-5.020	-7.949	-1.567	1000.000	0.004	**

Random effects				
	$\sigma^2_{\text{post.mean}}$	2.5% CI	97.5% CI	eff.samp
population identity	2.7812	1.0072	5.2264	1000.0000

**(g) Maternal fecundity**						
Fixed effects						
	$\beta_{\text{post.mean}}$	2.5% CI	97.5% CI	eff.samp	P_{MCMC}	Sig.
intercept	-1.489	-2.187	-0.616	1000.000	0.001	***
parasite load*	-0.003	-0.016	0.007	1108.687	0.560	n.s.
maternal standard length	0.053	0.043	0.064	1000.000	0.001	***
proportion maternal body fat†	-0.666	-1.478	0.085	1000.000	0.096	n.s.
proportion maternal body fat^{2}‖	-0.021	-0.067	0.026	818.957	0.434	n.s.
latest stage¶	0.031	0.024	0.039	897.857	0.001	***

Random effects				
	$\sigma^2_{\text{post.mean}}$	2.5% CI	97.5% CI	eff.samp
population identity	0.0832	0.0270	0.1640	1435.8285

**(h) Degree of superfetation**						
Fixed effects						
	$\beta_{\text{post.mean}}$	2.5% CI	97.5% CI	eff.samp	P_{MCMC}	Sig.
intercept	-0.000	-0.837	0.840	1000.000	0.994	n.s.
parasite load*	0.000	-0.015	0.016	1062.766	0.936	n.s.
maternal standard length	0.004	-0.009	0.016	896.360	0.574	n.s.
proportion maternal body fat†	0.003	-0.891	0.883	1000.000	0.966	n.s.
latest stage¶	0.020	0.009	0.029	680.016	0.001	***

Random effects				
	$\sigma^2_{\text{post.mean}}$	2.5% CI	97.5% CI	eff.samp
population identity	0.0086	0.0014	0.0189	1000.0000

**(i) Abortion incidence**						
Fixed effects						
	$\beta_{\text{post.mean}}$	2.5% CI	97.5% CI	eff.samp	P_{MCMC}	Sig.
intercept	0.172	-0.199	0.604	1000.000	0.388	n.s.
parasite load*	0.001	-0.006	0.008	1000.000	0.892	n.s.
maternal standard length	-0.002	-0.007	0.004	1000.000	0.546	n.s.
proportion maternal body fat†	0.465	0.028	0.878	1000.000	0.034	*

Random effects				
	$\sigma^2_{\text{post.mean}}$	2.5% CI	97.5% CI	eff.samp
population identity	0.0156	0.0031	0.0305	1000.0000

*number of black spots per female; †arcsin square-root transformed; ‡median-centered developmental stage of the most developed brood; §quadratic *z*-standardized developmental stage of the most developed brood; ‖quadratic *z*-standardized arcsin square-root transformed proportion of maternal body fat; ¶developmental stage of the most developed brood; $\beta_{\text{post.mean}}$: posterior mean of regression coefficient; CI: confidence interval; eff.samp: effective sample size; P_{MCMC}: posterior bayesian *p*-value; significant codes: $P_{\text{MCMC}} \leq 0.001^{***}$, $< 0.01^{**}$, $\leq 0.05^{*}$, > 0.05 n.s.

Table S7. Parameter estimates of the generalized linear model predicting variation in the probability of emerging from the box and crossing the metal ring. Whether an individual emerged from the box and crossed the metal ring was fitted as a binary variable (yes or no) using maximum likelihood and a logit link for the binomial-distributed response.

	Estimates				
	β	SE	*z*	*P*	Sig.
intercept	8.511	3.714	2.292	0.022	*
standard length	-0.153	0.089	-1.715	0.086	n.s.
males	0.806	1.216	0.663	0.507	n.s.
parasite load*	0.004	0.015	0.254	0.800	n.s.
day 2	-1.361	0.773	-1.761	0.078	n.s.

*number of black spots per female; β: regression coefficient; significant codes: $P < 0.001^{***}$, $< 0.01^{**}$, $\leq 0.05^{*}$, > 0.05 n.s.

Table S8. Parameter estimates of the generalized linear model predicting variation in the boldness score (i.e. time taken to emerge from the box) (s) fitted using maximum likelihood and a log link for the quasipoisson-distributed response.

	Estimates				
	β	SE	*t*	*P*	Sig.
intercept	3.153	1.120	2.814	0.007	**
standard length	0.013	0.028	0.448	0.656	n.s.
males	0.201	0.332	0.606	0.547	n.s.
day 2	0.970	0.300	3.232	0.002	**
parasite load*	-0.002	0.006	-0.372	0.712	n.s.

*number of black spots per female; β: regression coefficient; significant codes: $P < 0.001^{***}$, $< 0.01^{**}$, $\leq 0.05^{*}$, > 0.05 n.s.

Table S9. **Parameter estimates of the generalized linear model predicting variation in hesitancy (s) fitted using maximum likelihood and a log link for the quasipoisson-distributed response.**

	Estimates				
	β	SE	*t*	*P*	Sig.
intercept	2.198	1.293	1.699	0.097	n.s.
standard length	0.059	0.032	1.824	0.075	n.s.
males	0.584	0.315	1.853	0.071	n.s.
parasite load*	-0.002	0.006	-0.325	0.747	n.s.
day 2	0.434	0.263	1.650	0.107	n.s.

*number of black spots per female; β: regression coefficient; significant codes: $P < 0.001^{***}$, $< 0.01^{**}$, $\leq 0.05^{*}$, > 0.05 n.s.

References

[1] M. E. Brooks, K. Kristensen, K. J. van Benthem, A. Magnusson, C. W. Berg, A. Nielsen, H. J. Skaug, M. Mächler, and B. M. Bolker. glmmTMB balances speed and flexibility among packages for zero-inflated generalized linear mixed modeling. *The R Journal*, 9(2):378–400, 2017.

[2] K. K. Burnham and D. D. Anderson. *Model selection and multimodel inference: a practical information-theoretic approach*. Springer-Verlag, 2 edition, 2002.

[3] J. D. Hadfield. MCMC methods for multi-response generalized linear mixed models: the MCMCglmm R package. *Journal of Statistical Software*, 33(2):1–22, 2010.

[4] R. F. Hauer and G. A. Lamberti. *Methods in stream ecology*. Elsevier, Amsterdam, 2 edition, 2007.

[5] B. J. A. Pollux and D. N. Reznick. Matrotrophy limits a female's ability to adaptively adjust offspring size and fecundity in fluctuating environments. *Functional Ecology*, 25:747–756, 2011.
